# TadGAN-Based Daily Color Temperature Cycle Generation Corresponding to Irregular Changes of Natural Light

**DOI:** 10.3390/s22207774

**Published:** 2022-10-13

**Authors:** Seung-Taek Oh, Deog-Hyeon Ga, Jae-Hyun Lim

**Affiliations:** 1 Smart Natural Space Research Center, Kongju National University, Cheonan 31080, Chungcheongnam-do, Korea; 2 Department of Computer Science & Engineering, Kongju National University, Cheonan 31080, Chungcheongnam-do, Korea; 3 Department of Urban Systems Engineering, Kongju National University, Cheonan 31080, Chungcheongnam-do, Korea

**Keywords:** TadGAN, recursive TadGAN, natural light, CCT, daily color temperature

## Abstract

This study to develop lighting is advanced for reproducing natural light color temperature beneficial to humans. Methods were introduced to provide daily color temperature cycles through formulas based on the measured natural light characteristics or real-time reproduction of natural light color temperature linking sensors. Analysis results for the measured natural light showed that irregular color temperature cycles were observed for more than 90% of the year due to the influence of regional weather and atmospheric conditions. Regular color temperature cycles were observed only on some clear days. The color temperature cycle dramatically affects the health of the occupants. However, since irregular color temperatures are difficult to predict and cannot easily generate cycles, only the color temperatures of some clear days are currently used, and the actual color temperature of natural light cannot be reproduced. There is little research on deriving real-time periodic characteristics and lighting services targeting irregular color temperatures of natural light. Therefore, this paper proposes a TadGAN (Time Series Anomaly Detection Using Generative Adversarial Networks)-based daily color temperature cycle generation method that responds to irregular changes in the natural light color temperature. A TadGAN model for generating the natural light color temperature cycle was built, and learning was performed based on the dataset extracted through the measured natural light characteristic Database. After that, the generator of TadGAN was repeatedly applied to generate a color temperature cycle close to the change of natural light. In the performance test of the proposed method, it was possible to generate periodic characteristics of the irregular natural light color temperature distribution.

## 1. Introduction

Light is an essential environmental element for human survival and activity, and humans have lived for a long time by depending on the natural light environment [1,2]. Natural light shows a low color temperature and brightness at sunrise and sunset, but it continues to change in a pattern that increases during the day, significantly affecting the human body [3,4]. The human body’s intrinsically photosensitive retinal ganglion cells (ipRGCs) are directly involved in circadian rhythms by transmitting light information from the outside to the suprachiasmatic nucleus (SCN) [5,6]. Natural light also positively affects human brain wave activity and hormone secretion, providing the most ideal light environment for humans [7]. However, while lighting technology has been developed and modern people’s lifestyles have changed, exposure to indoor artificial lighting increased, and humans cannot enjoy the benefit of natural light [8]. Research cases that informed the physiological effects of lighting on the human body and the benefits of natural light exposure have been presented, and efforts to develop healthier lighting have continued [9].

Modern lighting technologies strive to construct a light environment close to natural light. Studies proceed to discover how to reproduce the wavelength and dynamically changing characteristics of natural light that is recognized as the best light source according to time [10]. Significantly, a technology to reproduce the natural light color temperature (CCT) cycle that keeps changing in a day and maintains biorhythm has been continuously attempted [11]. Nie et al. provided a color temperature of natural light at each time zone, like morning, evening, and noon, by controlling the LED lighting constituted of multiple channels [12]. Kim et al. collected and analyzed the spectral characteristics of natural light using a spectral radiometer, drew the color temperature cycle and wavelength characteristics from sunrise to sunset, and then provided it through LED lighting [13]. In addition, a method to provide a color temperature of natural light that kept on changing according to season or by one-year cycle compared with the daily natural light characteristics was introduced. Commercial products that realized such color temperature provision were also released [14]. However, the previous lighting technology could not provide a natural color temperature cycle that kept changing over the year.

In the research report which provided the status of sunlight reaching the ground, only 25% of days were classified as sunny (USA, largest cities, cloud covers 30 percent or less), and sunny days (cloud amount 1 or less) with an even distribution of color temperature of natural light in Korea were extremely low at 13% [15,16,17]. The color temperature of natural light shows a parabolic cycle only on a handful of clear days, and irregular color temperature cycles are formed on most days. The color temperature cycle influences the health of the occupants. Therefore, a lighting technology capable of stably providing a daily color temperature cycle is required to provide a realistic natural light color temperature, even when irregular patterns are mixed. However, since it is challenging to derive periodic characteristics when the irregular color temperature is measured due to the influence of weather or climate, only a part of the natural light color temperature cycle is currently provided [18,19]. Recently, a deep learning technique has been widely applied to detecting errors or extracting features for the time series data observed by time [20,21]. Deep learning was applied to monitor the change in the indoor light environment according to the effect of natural light over time. However, deep learning was executed only on clear days that were easy to predict [22]. In addition, the irregular color temperature within the color temperature cycle of natural light was defined as an anomaly, and LSTM was applied to detect it. However, it was implemented by limiting the color temperature cycle on clear days having stable time series patterns [16]. Although natural light exhibits repetitive periodic characteristics from sunrise to sunset, there is no case of applying deep learning to the color temperature of natural light, which is observed in most irregular patterns. However, there was no case in which deep learning technology was applied to the color temperature of natural light, which, although showing repetitive periodic characteristics with sunrise and sunset as criteria, exhibits different periodic characteristics at every moment of each day. TadGAN (Time Series Anomaly Detection Using Generative Adversarial Networks) can specifically give a pattern output similar to the original input through the generator; however, there is no instance of applying the generator to the irregular color temperature pattern of natural light. Studies on the derivation of natural light color temperature cycles are generally very scarce.

This paper proposes a method to generate a daily natural light color temperature cycle using TadGAN (Time Series Anomaly Detection Using Generative Adversarial Networks) even when the real-time color temperature is irregular due to changes in weather and atmospheric conditions. First, a natural light characteristic DB (Database) was built by measuring and collecting the characteristics of natural light that changed over time through a spectroradiometer. Afterward, a learning dataset was constructed by extracting and connecting the daily color temperature cycle values that exhibited regular color temperature distributions (anomaly ratio of 15% or less) through analysis, and then a training dataset was configured. Subsequently, an initial model of TadGAN that generated natural light color temperature cycles was built, and learning was executed. The generator function of the TadGAN model was repeatedly performed to construct a recursive TadGAN model that could extract the color temperature cycle closest to the original color temperature of natural light. In addition, an experiment was conducted to apply the recursive TadGAN model to various measured natural light color temperature cycles to confirm the proposed method’s daily color temperature cycle generation performance.

## 2. Creation of a TadGAN-Based Daily Color Temperature Cycle

**Figure 1:**
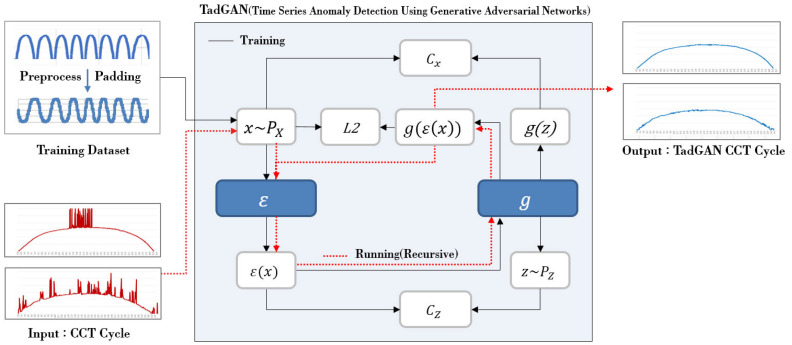
Generation of a TadGAN-based natural light color temperature cycle.

The color temperature of natural light is time series data that varies and is measured every moment. To construct the time series data of an accurate natural light color temperature cycle, color temperatures for 24 h are required. However, since the color temperature can be measured only between sunrise and sunset during the day, it is not easy to construct daily cycle time series data. Therefore, in this study, a padding feature was applied to construct data for learning and processing natural light color temperature so that the day’s color temperature was reconstructed and combined for the duration when natural light was unavailable. In addition, TadGAN (Time Series Anomaly Detection Using Generative Adversarial Networks), which could generate a pattern close to the original for unspecified input values, was applied to generate periodic characteristics for the color temperature values of natural light that changed over time [23,24]. It aimed to generate a daily color temperature cycle by applying TadGAN, which repeatedly applied the generator (ε, g) for the input sequence of the real-time natural light color temperature, showing an irregular change pattern. Figure 1 shows the flow of generating natural light color temperature cycle by applying TadGAN [23].

As shown in Figure 1, TadGAN consists of two generator models (ε, g) and discriminator models ($C_{x}$, $C_{Z}$) [24]. The generator model e divides the natural light color temperature values, which are the input domain (*X*), into a time series sequence ($x\sim P_{X}$) of a specific window size, and then converts it into a latent variable (*Z*). After that, the generator model g decodes the latent variable (*Z*) into time series data. Based on the results ($z\sim P_{Z}$), TadGAN gives the output of reconstructed time series data of the color temperature. In addition, the discriminator model $C_{x}$ distinguishes the original and reconstructed time series data, and the discriminator model $C_{Z}$ determines the degree of mapping of latent variables. At this time, the similarity degree of the reconstructed time series data with the original is calculated using the *L*2 norm as a loss [25]. In the proposed method, the reconstruction function of the TadGAN was utilized. After extracting the learning data set from the measured natural light color temperature data, the TadGAN learning was executed, and then the generator model of the TadGAN was repeatedly applied. The above procedures were intended to generate a daily color temperature cycle even when the color temperature of natural light by time was irregular due to changes in weather or atmospheric conditions.

### 2.1. A Training Dataset of Natural Light Color Temperature

**Figure 2:**
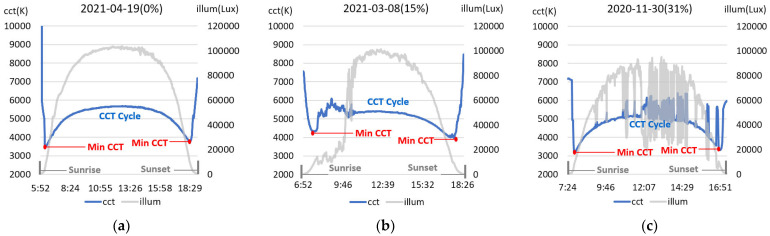
Example of daily color temperature cycle of natural light. (**a**) ‘21.04.19. (Anomaly ratio: 0%); (**b**) ‘21.03.08. (Anomaly ratio: 15%); (**c**) ‘20.11.30. (Anomaly ratio: 31%).

To learn the proposed TadGAN model, it was necessary to analyze the characteristics of the natural light color temperature cycle observed on the ground and extract the training dataset. For natural light color temperature characterization, natural light was measured for about one year (July 2020 to June 2021) through a spectroradiometer (CAS 140CT, Instruments, Germany) and a solar tracking facility at latitude 36.85 and longitude 127.14. The color temperature, illuminance, and spectral characteristics of natural light were measured at 1-min intervals for a total of 299 days, except for days that were difficult to measure due to the deterioration of weather conditions such as typhoons or monsoons. Figure 2 shows the changes in illuminance and color temperature between sunrise and sunset in the characteristics of natural light collected by actual measurement.

In Figure 2, the difference in color temperature for each minute was calculated to express the weather and atmospheric conditions for each measurement day. A case in which the difference in color temperature was more than 50 K was defined as an anomaly [16], and the rate of inclusion of the anomaly was indicated in the figure to estimate the degree of irregularity in the color temperature cycle on each measurement day. (a) corresponds to a sunny day with a precisely even distribution of the color temperature cycle, and the color temperature cycle of 23.8% (anomaly ratio 0–5%) of the total measurement days was similar. (b) is an example of the color temperature cycle for days with an anomaly ratio of 15%, with 25.8% of the total measurement days (anomaly ratio 5–15%) falling into this category. (c) is an example of a day with an anomaly ratio of 30%, and about 31.6% (15–30% of anomaly ratio) during natural light measurement days fell into this category. These days, many zones of irregular color temperature were observed. On the days of natural light measurement of about 17.5% (anomaly ratio 30–60%) and 1.4% (anomaly ratio more than 60%), the natural light color temperature cycle characteristics were not precise because the sky was cloudy or affected by the clouds. Furthermore, on all days in Figure 2, the illuminance at sunrise and sunset was the lowest, and the illuminance was the highest around noon. However, the color temperature’s lowest value ($CCT_{min}$) was observed within a particular time between sunrise and sunset, and a high color temperature distribution pattern around noon was observed. Based on the natural light color temperature characteristics analysis, the daily color temperature cycle was limited to the hourly color temperature value between the lowest color temperature ($CCT_{minAM}$, $CCT_{minPM}$) point after sunrise and before sunset. It was expressed as Equation (1).

(1) $$CCT_{cycle}=\left(CCT_{0},\;CCT_{1},\;CCT_{2},\;\ldots \ldots \;,\;CCT_{i}\right),\;\;i\;is\;time$$

$$CCT_{0}=CCT_{minAM}\;\mathrm{at}\;\mathrm{Sunrise},\;CCT_{i}=CCT_{minPM}\;\mathrm{at}\;\mathrm{Sunset}$$

**Figure 3:**
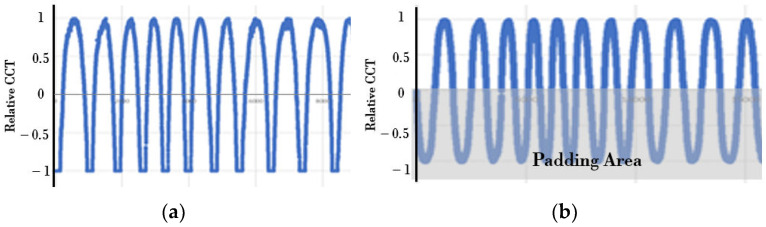
Color temperature training datasets (Anomaly ratio lower than 15%). (**a**) Training dataset—Original; (**b**) Training dataset—After implementing a padding.

To implement the TadGAN model that generates the color temperature cycle of natural light, it is necessary to build a training dataset consisting of daily color temperature cycle values that show an even distribution. The proposed method constructed a learning dataset using daily color temperature cycle values corresponding to 148 days with an anomaly ratio of less than 15%. The color temperature data with anomalies were included in the learning dataset to generate a color temperature cycle even when irregular color temperatures occurred. The training dataset was constructed in a way that connected the color temperature cycle values for each day. Figure 3 is an example of the training dataset.

Since the training dataset extracted and connected only the color temperature values between sunrise and sunset out of 24 h, the color temperature cycle for each day rapidly changed, as shown in Figure 3a. However, distortion of the color temperature cycle occurred during the training process in the connective section artificially configured to change the color temperature cycle rapidly in the TadGAN model, which used a time series sequence at regular time intervals as input and processing units. Therefore, padding was applied to the connective section of the color temperature cycle. When the padding was applied to express a smooth curve near sunrise and sunset, the color temperature cycle for each date was symmetrically inverted, and the padding of a reverse parabolic pattern connected in front of the original color temperature cycle of the day was applied. The result is shown in Figure 3b.

### 2.2. TadGAN Model That Generates Color Temperature of Natural Light

**Figure 4:**
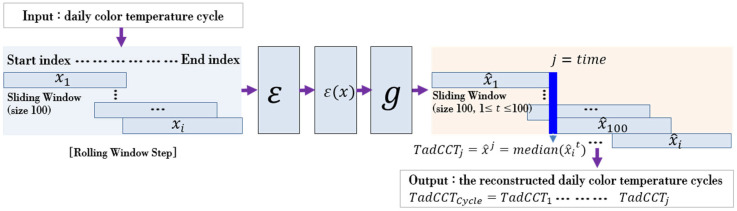
Color temperature generation process of TadGAN.

Training based on the previously constructed training dataset was executed to establish the TadGAN model in which the color temperature cycle of natural light was generated. After setting the sliding window size to 100 and constructing time series sequence data ($x\sim P_{X}$) at 100 s. intervals, the TadGAN was sequentially applied. The time series data of the color temperature were converted to the sequence $x_{i}^{1\ldots t}$(hereinafter expressed as $x_{i}\;$, where ‘i’ was the time series sequence number) having a specific size (t = 100), and then a reconstructed sequence ${\hat{x}}_{i}\approx g\left(\varepsilon \left(x_{i}\right)\right)$ having the same size through the processing of $x_{i}\to \varepsilon \left(x_{i}\right)\to g\left(\varepsilon \left(x_{i}\right)\right)$ was created. Therefore, a total of 100 reconstructed sequences were sequentially generated per hour. The median value for the $\;{\hat{x}}_{i}{}^{t}$ (where 1≤t≤100) values at a time (j) were taken and ${\hat{x}}^{j}=TadCCT_{j}$ was calculated. Figure 4 presents the natural light color temperature generation using TadGAN.

**Figure 5:**
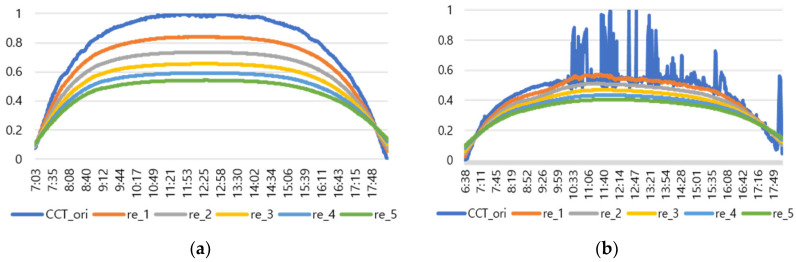
Experiment 2 by applying TadGAN: Generation of the TadGAN-based color temperature cycle. (**a**) Clear day (on 18 March 2020, 5 iterations of recursive model); (**b**) Cloudy day (on 14 September 2020, 5 iterations of recursive model).

After that, by applying the TadGAN until time corresponded to the end of the color temperature cycle, a reconstructed color temperature cycle ($TadCCT_{1},\;\ldots ,\;TadCCT_{j}$) was generated. The color temperature cycle generated through TadGAN showed a softer parabolic shape than the pattern of the original color temperature and produced a lower color temperature cycle than the original. Therefore, a color temperature cycle similar to the original could be generated if the heights of the color temperature cycle were adjusted for each section after removing the irregular color temperature pattern through the recursive application of TadGAN. Figure 5 shows the results of applying TadGAN recursively on clear days and cloudy days, respectively, and the results of recursively executing TadGAN till the fifth round are presented.

As shown in Figure 5, each time TadGAN was repeatedly applied from 1 time (re_1) to 5 times (re_5), a cycle curve with a lower color temperature than the input value was obtained. Therefore, it was judged that if weights were applied to the *N*th-order recursive result in the form of a curve, a color temperature cycle similar to the original would be generated, so the following recursive TadGAN method was implemented. Equation (2) is the output result of the TadGAN for each recursive count at each time (*i*).

ε : X → Z and g : Z → X

(X is input Domain, Z is latent Domain).

$$X={\left\{\left(CCTseq_{i}^{1\ldots t}\right)\right\}}_{i=1}^{N},\;CCTseq_{i}^{1\ldots t}\in \;X,\;x_{i}\to \varepsilon \left(x_{i}\right)\to g\left(\varepsilon \left(x_{i}\right)\right)\;\approx \;\hat{x_{i}}\;$$

(2) $$reN\_TadCCT_{i}=f_{median}\left({\hat{x_{i}}}^{t}\right),\;\left(i=index\;of\;TadCCT,\;1\leq t\leq 100,\;j=time\right)$$

When the TadGAN was applied *N* times recursively, the color temperature ($reN\_TadCCT_{i}$) of the TadGAN for each iteration was generated at every moment. Since it was confirmed that TadGAN generated a lower color temperature value than the previous iteration each time of applying recursively, an increased rate ($RoI_{j}$) and a scale constant (sc_K) at each moment were applied to correct this. The instantaneous increase rate was calculated through Equation (3).

(3) $$RoI_{j}=\frac{reN-1\_TadCCT_{j}}{reN\_TadCCT_{j}}$$

Rate of increase (RoI, instantaneous increase rate) is the ratio of the average color temperature difference between the output of the color temperature sequence ($TadCCT_{j}$) at each time (*j*) after applying TadGAN for *N* iteration and the output of the recursive TadGAN at the previous iteration (*N*-1 iteration). When the iteration of the TadGAN application exceeded 3, the color temperature from the actual color temperature increased near sunrise and sunset (Figure 4). It was confirmed through the preliminary experiment that when RoI at *N* = 3 was applied, the TadGAN model showed the best performance. Therefore, the recursion was set to 3, and the RoI at this time was calculated and applied. In addition to the color temperature difference at every moment, a scale constant was applied to correct the height of the overall color temperature cycle curve. The scale constant was calculated by applying Equation (4) to the color temperature sequence for the first 100 s.

(4) $$\;\;MAE_{K}=\frac{1}{n}\overset{n}{\underset{j=1}{\sum }}\left|realCCT_{j}-reN\_TadCCT_{j}\times RoI_{j}{}^{K}\right|,\;\;1.1\leq K\leq N+1$$

$$\;\;\;MAE_{min}=f_{min}\left(MAE_{1.1},\;MAE_{1.2},\;\ldots ,\;MAE_{K},\;\ldots ,\;MAE_{N+1}\right)$$

$$\;\;\;sc\_K=K,\;if\;MAE_{min}=MAE_{K}$$

As shown in Equation (4), the average absolute error with the original color temperature period was calculated after multiplying the application result of the TadGAN for each recursion iteration by the K-th power of the instantaneous increase rate. After calculating the mean absolute error by gradually increasing K by 0.1, K with the minimum mean absolute error was selected as the scale constant. The formula for calculating the real-time color temperature at every instant by applying the scale constant calculated through Equations (3) and (4) is shown in Equation (5).

(5) $$\;\;TadCCT_{j}=reN\_TadCCT_{j}\times RoI_{j}{}^{sc\_K}$$

$$\;\;\;TadCCT\_Cycle=\left(TadCCT_{1},\;TadCCT_{2},\;\ldots \ldots ,\;TadCCT_{j}\right),\;j\;is\;time$$

The color temperature was calculated every hour through the proposed recursive TadGAN using Equation (5), and the daily color temperature cycle (TadCCT_Cycle) was created by connecting them.

## 3. Experiments and Analysis

For the performance verification of the proposed method, a color temperature cycle that could be a standard and a color temperature cycle for each ratio, including each anomaly for comparison, were required. First, 3 sunny days with an anomaly within 2% were selected, and anomalies of 10%, 30%, and 60% ratios were randomly generated and combined. Table 1 shows the results of applying the recursive TadGAN proposed to the color temperature cycle for each anomaly inclusion ratio.

In Table 1, the “Category” column is the ratio of anomalies to the original color temperature cycle. The original color temperature cycle is real natural light color temperature cycle collected through measurement. The original color temperature cycle was compared before applying the TadGAN (results including anomalies), standard TadGAN, and the recursive TadGAN proposed in this study. In general, when the anomalies were included in the original, the distribution of color temperature cycles was irregular, and when the standard TadGAN was applied, it was possible to generate color temperature. However, a zone with a significant difference from the original color temperature cycle was observed, and the difference between sunrise and sunset seemed more significant than when the recursive TadGAN was applied. Table 1 (a) showed the result of applying the proposed method after including 10% of anomalies in the daily (29 April 2020) color temperature cycle. When the anomalies was included, the mean absolute error (MAE) of the color temperature was 64.9 K, and when the standard TadGAN was applied, the MAE was 21.7 K. Whereas when the recursive TadGAN was applied, the MAE was 13.9 K. Even when the rate of inclusion of anomalies in Table 1 (b) was 30%, the MAEs before TadGAN, after standard TadGAN, and in the results of applying recursive TadGAN were confirmed to be 124.7 K, 26.9 K, and 26.5 K, respectively. In addition, even when the anomalies inclusion ratio in Table 1 (c) was 60%, the MAE of the result of applying the recursive TadGAN was 53.2 K. Therefore, it was proven that the recursive TadGAN could generate a better color temperature cycle than before applying TadGAN (259.3 K) and standard TadGAN (66.1 K). However, in the results of Table 1 (b), the performance was similar to that of the existing TadGAN according to the distribution of color temperature anomalies. When the anomaly inclusion ratio was adopted, it was possible to generate a natural color temperature cycle within the range of MAE 26.5 K for around 81.2% days (anomaly ratio within 30%, 242 days) among measured natural light days of 299 in a year, and within MAE 53.2 K for about 98.7% days (292 days) in a measured natural light dataset with an anomaly ratio within 60%.

**Figure 6:**
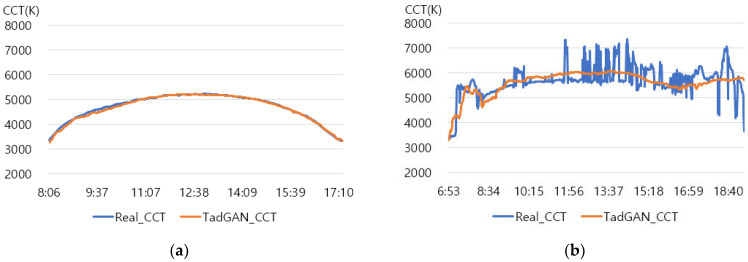
TadGAN-based color temperature cycle generation experiment. (**a**) Clear day: anomaly ratio less than 5%; (**b**) Cloudy day: anomaly ratio more than 30%.

An experiment was conducted to check the possibility of generating a color temperature cycle through the proposed method to input the actual natural light color temperature. For the experiment, a sunny day with an anomaly ratio of less than 5% and a cloudy day with an anomaly ratio of about 30% were selected. Figure 6 shows the results of applying the proposed method.

In the result of (a) on a sunny day (27 January 2018), it was found that the proposed method produced a color temperature cycle almost similar to that of the original. (b) is the result of a cloudy day (13 August 2019) where substantial irregularly colored temperature was measured due to the weather and atmosphere effect. Despite the unstable color temperature distribution of the original, the color temperature cycle of a particular pattern could be generated in real-time through the proposed method. Table 1 showed that the MAE of 31.2 K was improved by 80% compared to the original color temperature cycle, including anomalies. Therefore, creating a color temperature cycle was possible corresponding to the irregular natural light color temperature change. It was also possible to apply the proposed method to the real-time measurement of natural light.

## 4. Conclusions

Current lighting technology cannot reproduce a realistic natural light color temperature cycle because it only provides natural light color temperature focusing on clear days. This paper proposed a TadGAN-based daily color temperature cycle generation method corresponding to the irregular color temperature observed on most days of the year. First, for the training dataset of the TadGAN model, the color temperature for 136 days of natural light was collected and analyzed for about one year. After extracting the color temperature cycle values on a sunny day with an anomaly ratio of less than 15%, an inverse parabolic padding was applied, and a training dataset was constructed by connecting them. Afterward, a TadGAN was built to process a color temperature time series sequence of 100 s, and training based on the training dataset was executed. The learning of the color temperature data, including anomalies, was executed through TadGAN to generate a color temperature cycle even when an irregular color temperature occurred. The TadGAN was designed to generate a reconstructed color temperature cycle through two generators (ε, g). The color temperature cycle generated through the TadGAN model had a difference in height from the original cycle, showing a different pattern near sunrise and sunset. To improve this, a recursive TadGAN model that derived periodic characteristics like the original color temperature was constructed by repeatedly applying the generator of TadGAN. In the proposed method, the generator was repeated for three times, and the instantaneous increase rate and scale constant was applied to the result of the recursive TadGAN at each moment to generate the daily color temperature cycle. After that, anomalies of 10, 30, and 60% ratios were generated in a color temperature cycle of 3 days on a randomly selected sunny day, respectively. A comparative experiment was then performed to apply the existing TadGAN and the proposed recursive TadGAN. The results showed that the mean absolute error (MAE) of the color temperature was 31.2 K, which was about 80% improved compared to the original 149.5 K, exhibiting a color temperature cycle close to the original color temperature cycle, which indicated that it was possible to generate a real-time color temperature of natural light.

The ultimate purpose of this study is to provide indoor areas with the same light by tracking the color temperature of natural light in real time. In the future, additional experiments should be conducted to improve color temperature generating functions according to the distribution patterns of color temperature anomalies. In addition, follow-up studies are planned to confirm and process the start and end of the color temperature cycle for real-time application of the proposed model. In addition, efforts will be continued to develop a lighting system having natural light through linkage control with LED lighting.

## Figures and Tables

**Table 1:** Application results by applying the proposed TadGAN.

Category	Color Temperature Cycle (Including the Original and Anomalies)	Standard TadGAN	Proposed (Recursive) TadGAN
(a) Anomaly Ratio 10%	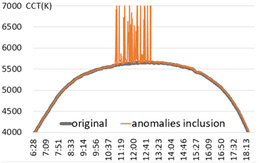	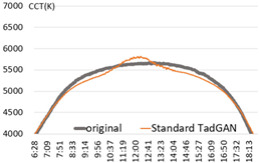	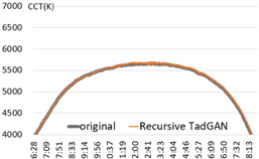
MAE	(29 April 2020) 64.6 K	21.7 K	13.9 K
(b) Anomaly Ratio 30%	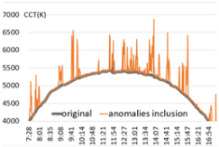	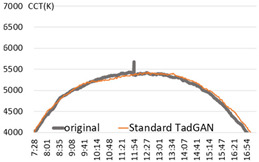	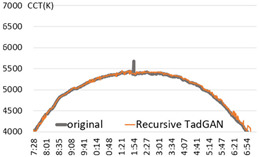
MAE	(20 October 2019) 124.7 K	26.9 K	26.5 K
(c) Anomaly Ratio 60%	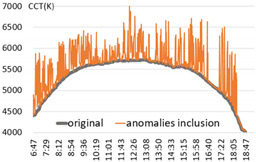	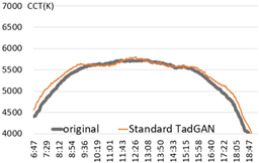	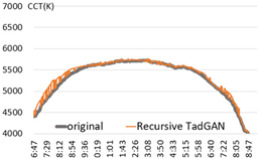
MAE	(21 July 2018) 259.3 K	66.1 K	53.2 K
Average	149.5 K	38.2 K	31.2 K

## Data Availability

Not applicable.
